# Challenges and new opportunities in deciphering the meaning of corvid call sequences

**DOI:** 10.1007/s10071-025-02015-3

**Published:** 2025-11-26

**Authors:** Ambre Salis, Killian Martin, Cédric Girard-Buttoz

**Affiliations:** 1 Department of Life Sciences, Imperial College London, Ascot, UK; 2 Ecole Normale Supérieure de Lyon, Lyon, 69007 France; 3 ENES Bioacoustics Research Laboratory, Centre de Recherche en Neurosciences de Lyon, CNRS, Inserm, University of Saint-Etienne, Saint-Etienne, France; 4 Department of Human Behaviour, Ecology and Culture, Max Planck Institute for Evolutionary Anthropology, 04103 Leipzig, Germany

**Keywords:** Animal communication, Corvid, Semantic, Meaning, Methodology

## Abstract

Due to their complex social systems and remarkable cognitive abilities, corvids are interesting candidates for large scale comparative research on the meaning of animal calls. However, research on corvid communication has primarily focused on individual signatures or mimicry capabilities, and investigations into the meaning of their calls have yielded comparatively fewer results. This discrepancy can be attributed to several challenges faced by researchers, including difficulties in identifying the units that convey meaning, accurately determining the specific context associated with a call, and the limitations of traditional playback methods when applied to species with extensive repertoires and considerable flexibility in call sequences. In this review, we outline a series of emerging research avenues—recently explored in other songbirds and mammals—that may prove valuable for researchers seeking to understand the meaning behind corvid call sequences. We specifically address the various approaches to identify meaning-bearing units; the strategies for refining the definition of ‘context’ in the assessment of corvids’ repertoires; and the novel protocols and methods that offer alternative perspectives on meaning, beyond the classical playback experimental approaches that were historically used to assess the meaning of calls or call sequences.

## Introduction

Comparative research is a highly effective tool for examining the theoretical foundations of the evolution of communication. By analysing the mechanisms of production and reception of acoustic signals across various species, then retracing the phylogenetic history of such traits, it becomes possible to distinguish between traits inherited through shared ancestry and those resulting from convergent evolution (i.e., the same trait emerged several times independently). This differentiation is valuable for discussing broad-scale evolutionary theories concerning the ecological and evolutionary pressures that have shaped the current diversity of animal communication. Due to increased and facilitated collaborations among international research teams in the last decades, alongside the development of advanced analytical techniques and shared methodologies, comparative research is experiencing considerable growth (e.g., based on Web Of Science, approximately 1300 in Zoology mentioned the term “Comparative research” in 2024, compared to only 400 in 2001). This comparative approach can, for instance, allow the investigation of large-scale statistical patterns, such as linguistic laws (Semple et al. 2022), and reveal that most species share similar pressures for both brevity and maximising transmission success (Bentz and Ferrer-i-Cancho 2016). Additionally, comparative studies are employed to test hypotheses explaining the evolution of complex communicative systems, such as the social complexity hypothesis, which posits that increasingly intricate social structures drive the development of more complex acoustic signalling (Freeberg et al. 2012; Peckre et al. 2019). From a cognitive perspective, comparative research on acoustic systems is instrumental in exploring the presence of specific cognitive mechanisms. For example, the concept of categorization (i.e., process by which an animal’s perceptual system sorts stimuli that vary in a continuous fashion into a set of discrete categories’, Green et al. 2020) has been extensively examined in several distantly related species, demonstrating that categorization may represent a fundamental cognitive operation underpinning acoustic processes in several animal species.

One specific part of comparative research that has recently received much attention is the concept of meaning. Meaning is a multifaceted concept that requires careful definitions to allow comparisons between studies and species. There is a long-standing debate that is still ongoing about the concept of meaning in animal communication (Hockett 1959; Berthet et al. 2023; Fischer and Price 2017; Seyfarth and Cheney 2017). A recent framework proposed by Amphaeris et al. (2023) suggests that meaning can be decomposed into three facets: the *signal* meaning facet, that relates to the meaning of the signal itself when produced by the caller, the *interactant* meaning facet that relates to the meaning that can be derived from the signal in the context of the social interaction between the caller and the recipient, and the *resultant* meaning facet where meaning is derived based on the outcome. While all three aspects must be considered, some are arguably easier to assess empirically than others in non-human animals. The framework by Amphaeris et al. builds on the historical definition of meanings in animal communication literature and, in particular, expands from the concept of functional referential signals that has been criticized for its limitation in interpreting the information content of animal vocalizations (Wheeler and Fischer 2012). Indeed, previous work has focused on the concept of referential signals in the attempt to draw an analogy between signals providing referential information in human language (words or sentences that refer to something in the mind or in the environment of the two interactants, Glock 2012).

It is extremely challenging to probe the minds of non-human animals to assess what they think and how signals are perceived in their mind. One strategy is to design playback experiments to trigger adaptive behavioral reactions in response to a signal but in the absence of the external stimuli that elicit the signal production (e.g., predator presence). This method has allowed previous studies to infer that some signal refers to something specific in the environment (Seyfarth et al. 1980; Sievers and Gruber 2016; Slocombe et al. 2022; Zuberbühler and Wittig 2011). This approach is, however, limited in its capacity to grasp the different facets of meanings and also fails to test certain meanings (e.g., a call that functions to prolong resting, indicating to others to “stay put”, can hardly be tested using playbacks). Playback usage can also become limited when it comes to assessing the meaning of all the calls in a large vocal repertoire, or when trying to assess the meaning of call sequences (see section *Playbacks* for more details). Recent exchanges about meaning among the fields of linguistics, ecology, ethology, and cognitive sciences have facilitated the development of new methods. For example, Berthet et al. (2023) propose that we could assess meaning as the *“set of features of circumstances (FoC) that appear at a rate greater than chance across the signal’s occurrences”*. By recording events surrounding the production of the vocalization, both external events (such as the presence of food or predator) but also social interactions co-occurring with (or resulting from) the signal production, one can match an event to a vocalization and infer the potential meaning of this vocalization without needing to conduct playbacks.

Because of their close genetic relatedness to us, both empirical studies and discussions largely focused on the meaning of calls (single or in combinations) in Primates (Engesser and Townsend 2019; Seyfarth and Cheney 2017; Fischer and Price 2017). Few studies provide such an assessment in birds despite noting their communicative potential (see e.g. Marler 2004; Suzuki et al. 2016; Engesser et al. 2016), and among birds, corvids have been less extensively studied in this domain. This is surprising since Corvids may constitute an interesting study taxa for comparative research to retrace the evolutionary origins of complex communicative systems in general and language in particular. Corvidae encompass a wide variety of species (128 across 21 genera, including crows, ravens, magpies, rooks, jays, and jackdaws) with habitats spanning across most of the world (all continents except Antarctica), and exhibit a wide variety of ecological niches, social organisations, and behavioural ecology (see e.g. Clayton and Emery 2007; Miller et al. 2022; Madge and Burn 1994). This diversity makes *comparative research within the corvid clade* potentially valuable in disentangling the evolutionary pressures that have led to the emergence of specific communication traits. For example, corvids appear to be an ideal group to investigate whether complex social systems are necessarily linked to complex acoustic systems (i.e., the social complexity hypothesis, Wascher and Reynolds 2025; Wascher et al. *under review*), since the diversity of social systems is impressive in this clade (e.g., common ravens form territorial pairs, rooks and jackdaws are group-living and breed colonially albeit not cooperatively, magpies and some species of jays live in territorial groups, Clayton and Emery 2007; Goodwin 1986).

In addition, some corvid species may also hold significance for *large-scale comparative studies* because they share traits with distant taxa including parrots and both human and non-human primates, enabling the exploration of convergent evolution: First, most corvid species studied have shown excellent **cognitive abilities** (see e.g. Emery 2004; Lambert et al. 2019; Taylor 2014 for reviews) in a wide variety of tasks (e.g. tool use and tool fabrication; delayed gratification; episodic-like memory; theory of mind). In particular, they have demonstrated the potential to control their behavior, such as by controlling call production (Brecht et al. 2019; Liao et al. 2024), or in deciding whether a delayed reward is worth the wait (Dufour et al. 2012), and generally remarkable memory capabilities (Boeckle and Bugnyar 2012; Marzluff et al. 2010; Taylor 2014). This suggests that some corvid species may be less constrained by the cognitive load limitations proposed to affect most animal species (Lind and Jon-And 2024), making them intriguing candidates for exploring shared cognitive and communication mechanisms across distant species. Second, many corvids are highly **social** species, often engaging in long-term and complex conspecific interactions (Clayton and Emery 2007). Certain species are capable of recognizing individual group members (Kondo et al. 2010), understanding relationships among them (e.g., recognition of ranks, Szipl et al. 2015). Some examples even hint for the ability to have Washburnian intention in ravens (i.e., the ability to manipulate other’s intentions, Scott-Phillips and Heintz 2023) since they can guard their caches against discovery, taking into account other ravens’ possible knowledge of the cache (Bugnyar et al. 2016). Finally, corvids appear to have complex **communicative** abilities. The most evident channel is through vocalizations, with most species exhibiting an impressive diversity of calls (Wascher and Reynolds 2025) and dedicating a significant portion of their time to vocalization. Corvids also use non-vocal ways of communication: for instance, ravens have been suggested to use specific movements to convey information, with similarities with human gestures (Pika and Bugnyar 2011), although this remains debated (Van Rooijen 2015; Pika 2016). Finally, several species use feather movements (e.g. ruffling or slicking head feathers) and particular body postures in social interactions (e.g. rooks Coombs 1960; jackdaws: Katzir 1983; magpies: Baeyens 1979). Since non-vocal communication is comparatively less studied than vocal communication in corvids, we focused on the latter in this review but acknowledge that gestural and multimodal communication are important as well. We aim hereby to encourage comparative research in the meaning of corvid calls that are highly promising due to the cognitive, social, and communication complexity of this taxon.

Some studies have indeed investigated the usage, function, and potential significance of corvid calls (see e.g. Wascher and Reynolds 2025 for an overview, and see *Playback* section for selected examples). However, these studies appear comparatively limited in scope relative to research conducted on other species (e.g., a Web of Science query conducted on 04/08/2025 with the keywords corvid + meaning yielded 144 studies, whereas primate + meaning resulted in 7893 studies). This lower number of studies examining the meaning of corvid calls can be attributed to a variety of factors. Even prior to determining potential meanings, the identification of corvids’ individual units of information (Kershenbaum et al. 2016) can already be highly challenging for many studies. In most passerine species, calls (defined as continuous traits on a spectrogram, separated by intervals of silence, Table 1) are often considered to be the units of information. Although continuous variations exist, in many species call classification can be performed both visually and through automated measurements with a high degree of repeatability (Kershenbaum et al. 2016). This first step then allows researchers to test simple hypotheses regarding the meaning of specific utterances containing specific calls. However, in corvids, some species produce continuous sequences, characterized by long durations with little to no pauses, which can make the identification of distinct units challenging (e.g., Brown 1985; Goodwin 1955; Lorenz 1970; Martin et al. 2024; Thompson 1982). Moreover, the classification of calls is complicated by the prevalent existence of a diverse and often graded repertoire, i.e., the frequent production of various intermediate forms between two distinct call types. To further complicate matters, some species exhibit minimal to no shared repertoires among individuals. For instance, male rooks seemingly share very few of their calls with neighboring individuals, even within the same social group (Martin et al. 2024). Several corvids have been described as producing large repertoires (e.g. 150 call types in White-throated Magpie-jays, Ellis 2008; 87 call types in Torresian crows, Brown 2011) or at least a large variety of functionally similar calls (e.g. 79 different “caw” call types in Northern ravens Enggist-Dueblin and Pfister 2002; over 30 in American crows, Yorzinski and Vehrencamp 2009). Determining the meaning of corvid vocalizations is further complicated by the acoustic structure of the vocalizations, as well as by the vocal production learning abilities of the corvid family. Corvid vocalizations often have highly chaotic acoustic structures (Fletcher 2000; Stowell et al. 2016), in particular with little harmonic structure. This chaotic acoustic structure makes corvid vocalizations much more difficult to analyze using classical, formant-based acoustic feature-driven methodologies that have been successfully applied to other species of birds, primates, or cetaceans. As a result, it is often unclear whether all individuals of a given species produce the same types of vocalizations. (e.g. Benti et al. 2019 had to exclude some rooks who did not produce acoustically recognizable “caw” calls despite specifically investigating individuality in this very call). Furthermore, corvids are open-ended vocal learners and can incorporate new sounds throughout their life (e.g. Brown 1985; Bluff et al. 2010; Wascher et al. this issue), whether through cultural transmissions of intra-specific vocalizations (e.g. Enggist-Dueblin and Pfister 2002; Kondo 2021) or by imitating the calls of other species (e.g. Brown 1985). A final difficulty is that contextual use can be challenging to interpret: observational studies in rooks revealed that most calls were not associated with a particular context (Martin et al. 2024), and the most frequent call types that were shared by several individuals were produced in multiple contexts (Benti et al. 2019; Røskaft and Epsmark, 1982). Finally, these difficulties are further compounded by the way corvid calls can be produced not only in isolation, but in sequence: this opens the possibility that information may be encoded not only in the calls produced, but also in their combination and/or repetition (see section ‘Focus on sequences’). An important distinction to make here is that corvids also produce songs, long sequences of vocalisations with great variety both between individuals and between renditions (Brown 1985; Martin et al. 2024). Unlike most other songbirds, corvid song is not involved in territorial defence or courtship, instead seemingly being produced outside of any obvious context (Brown 1985; Coombs 1960). This lack of context and variety suggest that corvid song does not convey specific information, and as such, distinct renditions would not have distinct meanings (Marler 1998; Rohrmeier et al. 2015; Pika 2017). For this reason, we excluded corvid song from consideration in this review, though several of the analytical techniques described below could apply to its study.

**Fig. 1:**
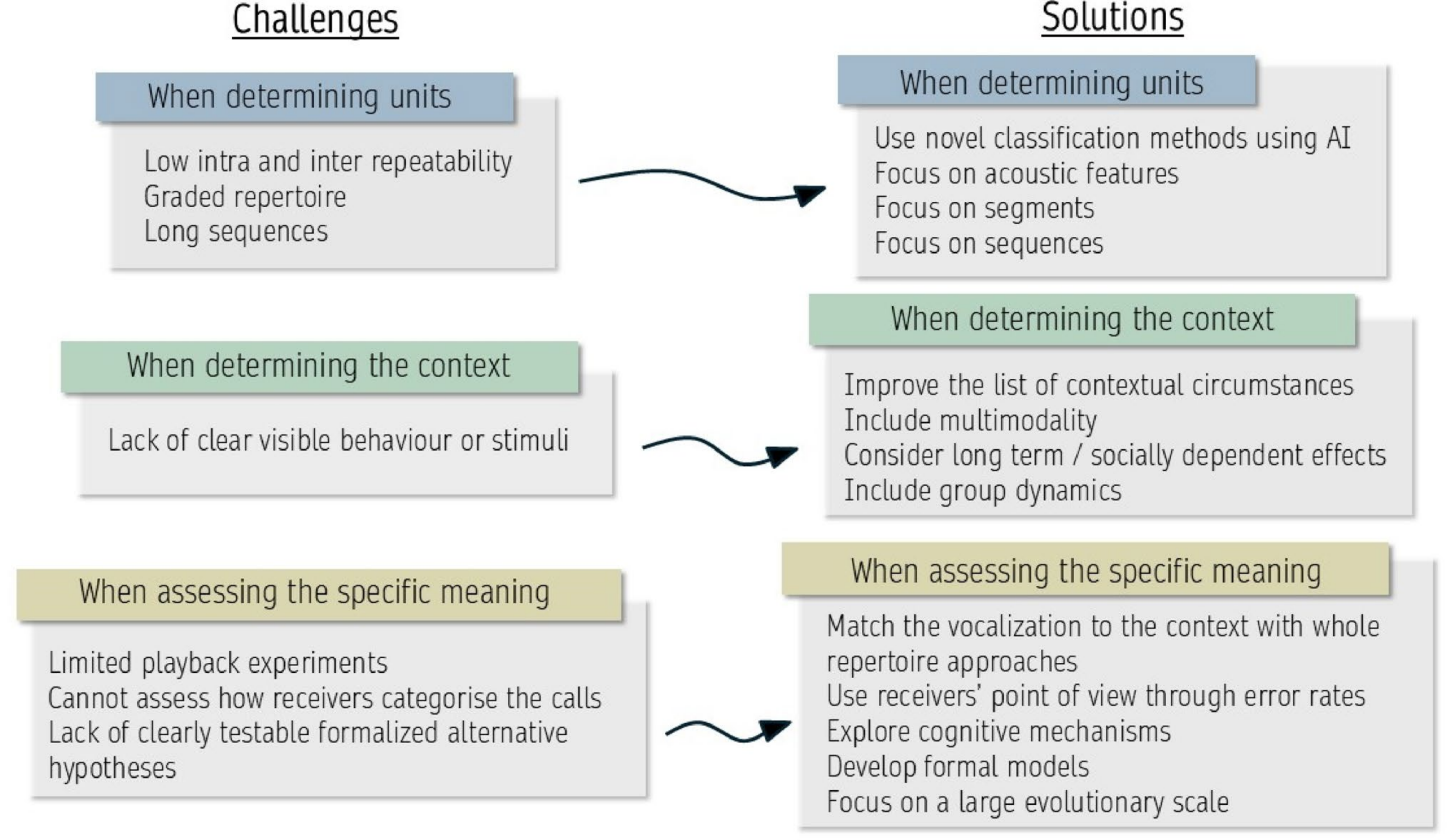
Common challenges encountered in corvid acoustic research and possible ways, implemented in other taxa, to solve these issues. All the propositions presented on the right part of the figure are expanded in more detail in the core text of the manuscript

In this manuscript, we do not aim to review the literature on corvid communication, nor do we delve into the reasons why linking acoustic and cognitive mechanisms (e.g., vocal learning) within this clade may be of particular interest, as both topics have already been thoroughly addressed in Wascher and Reynolds 2025 and Liao et al. 2025. Instead, to foster greater exchange between historically distinct fields, we present selected examples of methods and concepts that have been successfully applied to non-corvid species, with the hope that this will inspire future comparative research. We emphasize how these methods may be beneficial for certain corvid species, while acknowledging the inherent limitation that, given the extensive diversity of corvid ecology, not all methods are applicable to every species. Specifically, we highlight potential solutions at three key stages of any project aimed at elucidating the meaning of calls: the identification of **units**, the determination of the **context** surrounding a call sequence, and the specific procedures and statistical approaches that facilitate the exploration of **meaning** in animal communication. Fig. 1

**Table 1:** Glossary of terms. *These definitions are open to discussion*,* but will be the ones used in this review*

Vocalization	A generic term for a vocal output separated from other vocal outputs by long silent intervals.
**Call**	Part of a vocalization separated from other parts by short periods of silence before and after (Fig. 2).
**Call type**	Calls with specific acoustic features that are distinguishable from those of other call types.
**Segment**	A fraction of a vocalization with the same acoustic characteristic (Fig. 2)
**Call sequence**	A vocalization containing at least two calls (from the same or different call types) (Fig. 2)
**Meaning-bearing unit**	The smallest fraction of a vocalization that is meaningful – either an acoustic feature, a segment, a single call, or a sequence of different calls (e.g., A, B, and C in the Japanese tits ABC_D sequence, see text)
**Call combination**	A meaningful combination of at least two different meaning-bearing units

## Determine the meaning bearing units

**Fig. 2:**
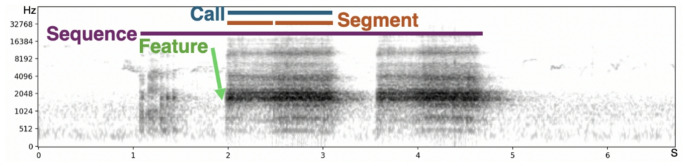
Example of a spectrogram of a call sequence from a rook (*Corvus frugilegus*) (own data from KM), to emphasize the possible ways to define meaning-bearing units. The horizontal axis represents time in seconds, the vertical axis represents frequency (in Hz, on the Mel scale), and the color scale represents sound amplitude (low: white; high: black). The blue horizontal line indicates the delineation of a call, a continuous trace on a spectrogram, separated from other calls by periods of silence. The orange horizontal lines delineate two segments that compose the call. Segments are clear variations in acoustic properties within the same call. The green arrow points at the presence of a specific acoustic feature, that is a specific acoustic property (in this example, the presence of a peak of amplitude at approximately 1500 Hz, but this can be any other feature, including temporal ones). The purple horizontal line delineates a call sequence composed of three calls from two different call types (see also Table 1 for definition of all the terms)

### Individual calls as the meaning bearing units: new methods to determine the repertoire

The simplest case of meaning-bearing units are the calls: vocalizations separated by silent intervals (Table 1; Fig. 2). They are often the simplest units to classify into categories, because they are easier to separate from each other (Kershenbaum et al. 2016). Identifying these calls is often the first step in studying the vocal repertoire. Classical methods based on manual inspection, or acoustic feature-based clustering, have been sufficient to determine the repertoire of several species (e.g. Knörschild et al. 2010; Elie and Theunissen 2016; Wadewitz et al. 2015). In the simplest case, all individuals produce the same set of distinct calls, and the meaning of each call can then be studied.

In species like the corvids, determining the catalogue of calls is much more challenging. Classical methods are often unsuited to clustering calls in these species because of their large vocal repertoires, chaotic vocal structure and graded vocalizations making calls difficult to categorize (Fletcher 2000), and high inter-individual variability (Martin et al. 2024). Historically, this has also limited many studies of corvid vocalizations to single vocalization type (essentially one call) or to the investigation of vocal signatures (e.g. Boeckle et al. 2012; Mates et al. 2015; Stowell et al. 2016; Yorzinski et al. 2006), but modern machine learning approaches may offer robust tools to catalogue corvid vocalizations and determine the clusters of calls needed to investigate corvid communication in more depth.

**Fig. 3:**
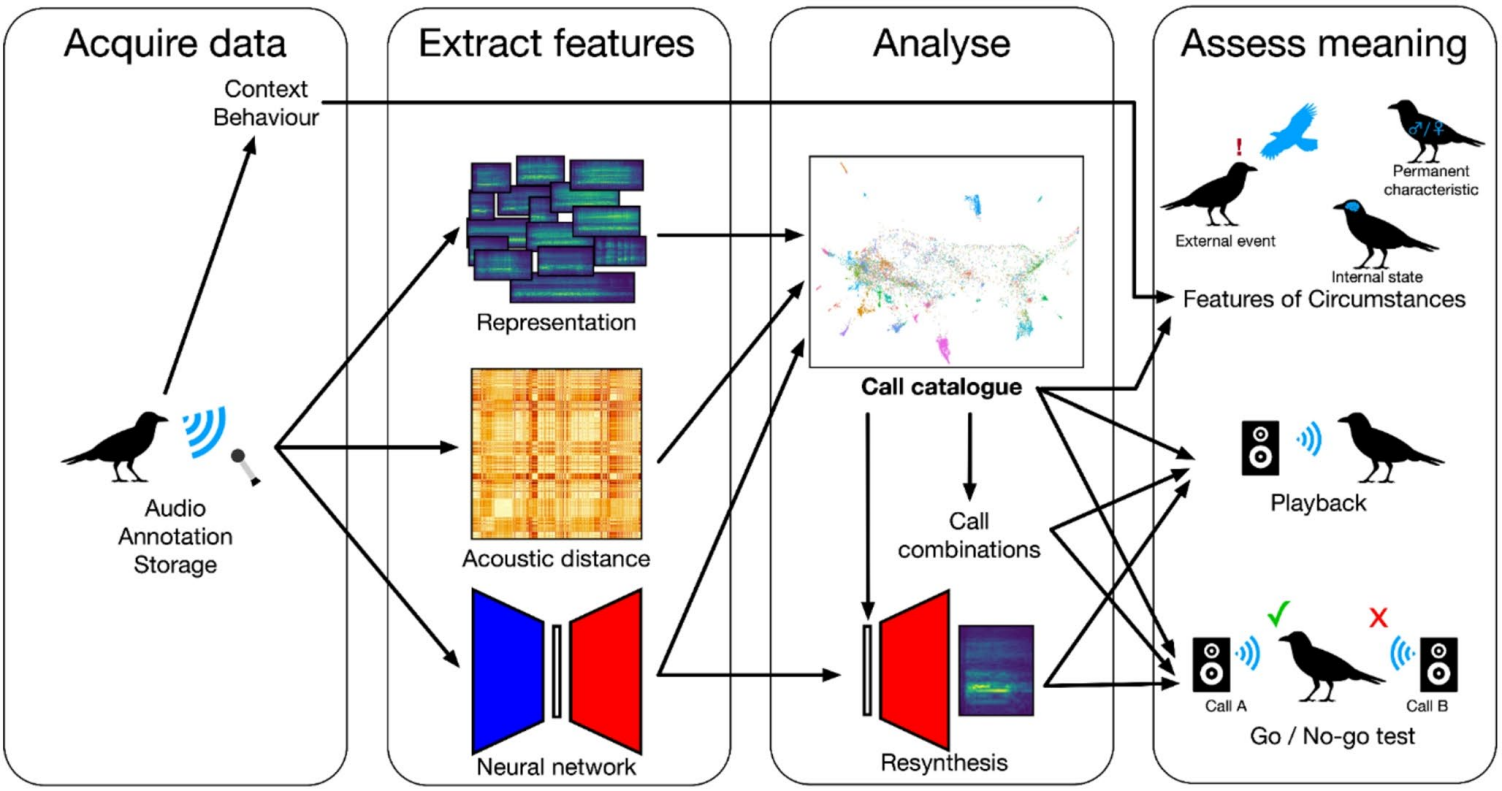
Illustrated workflow for constructing a catalogue of different calls and beginning experiments to determine meaning-bearing units. Specific sections of this article detail various parts of the workflow: the feature extraction stage and the construction of the call catalogue; the call resynthesis and general playback experiments to determine potential meaningful features; the meaning assessment using features of circumstances or with go/no-go experiments

Modern approaches in bioacoustics have increasingly relied on complete representations such as the spectrogram to determine clusters of vocalizations (see e.g. Best et al. 2023; Cohen et al. 2022; Kather et al. 2025; Martin et al. 2024; Pagliarini et al. 2021; Sainburg et al. 2020). Using the entire spectrogram provides much more information and removes some of the biases of other feature-based approaches, which allows much finer classification than with classical methods. Many of these approaches also leverage deep learning neural networks for complex pattern recognition. A staple of many neural networks is a bottleneck somewhere in the architecture of the network where representations of the vocalizations (often called “embeddings”) can be extracted and analyzed. Other approaches may base the feature extraction on computing distances between spectrograms, such as the Euclidean distance (Sainburg et al. 2020), spectrogram cross-correlation (Cortopassi and Bradbury 2000; Sawant et al. 2022), or dynamic time warping (Martin et al. 2024; Meliza et al. 2013; Somervuo 2019; Fig. 3). Regardless of the approach, clustering algorithms can be used to group vocalizations by acoustic structure, thus forming the basis of the catalogue of calls.

### Focus on features

When methods attempting to delineate calls as the meaning-bearing unit fail, one alternative approach is to shift our focus from treating calls as the units of information to considering the acoustic features within them as the factor impacting meaning (Aubin et al. 1989, Schlenker et al. 2025). In this view, it is the presence of one specific acoustic feature (spectral or temporal), that is conveying meaning to the receivers (Fig. 2). In other words, although several calls may appear distinct in a spectrogram analysis, if they share a specific acoustic feature, they may convey the same meaning. This view may be particularly useful in call sequences that, at first glance, show the repetitions of varying calls: subtle acoustic variations, difficult to detect by eye, may convey important information to receivers. Alternatively, features may serve to modify the meaning of a general call rather than embodying a specific meaning in themselves (e.g., it is not the presence of a peak frequency at 2500 Hz that is important, but rather the relative height of some calls compared to others). Consequently, an apparently homogenous call category may, in reality, conceal a diversity of meanings. For example, in a large range of species the inclusion of non-linear phenomena in a call typically indicates highly arousing situations and in particular situations with negative valence, such as aggression (Blumstein and Récapet 2009; Fournier et al. 2025). Other acoustic features such as pitch (Ficken 1990) or amplitude (Gustison and Townsend 2015), can also modulate the meaning of a general call. Finally, temporal features such as call rate and general rhythmic patterns can provide information. For example, a contact call repeated with a small inter-call interval changed to an alarm call in chaffinches (Randler and Förschler 2011). In northern elephant seals, identity coding can also be done through temporal features and recognized by conspecifics (Mathevon et al. 2017). Isochrony (when time intervals between successive onsets of a signal all have roughly equal durations, Ravignani and Madison 2017) is another promising lead, explored in most clades (insects, birds, mammals, frogs, Ravignani and Madison 2017).

The exploration of acoustic features is well established in the bioacoustics literature, via call resynthesis. To investigate this, two complementary approaches can be used. The first is a top-down approach, wherein a signal is modified until no behavioral response is elicited from a specific receiver (for example, modification of spectral and temporal features of the chick-a-dee call of the black-capped chickadee *Parus atricapillu*s had a direct impact on the level of response from the birds, Charrier and Sturdy 2005). The second is a bottom-up approach, in which an artificial call is constructed, utilizing the minimally hypothesized features believed to be the meaning-bearing units (for a recent example on great tits’ mobbing calls, see Salis et al. 2024). The combination of these two methods can lead to a very precise understanding of coding and decoding mechanisms in animals. For instance, a comprehensive analysis of king penguin calls has demonstrated that a specific modulation of frequency in the initial portion of the call is responsible for individual recognition (Jouventin et al. 1999). This focus on acoustic features has, to our knowledge, seldom been explored in corvids, and even less frequently within meaning-focused studies. Corvid vocalizations may be difficult to exploit in this manner, due to their chaotic structure (Fletcher 2000; Stowell et al. 2016) making many features difficult to isolate, let alone manipulate. Recent developments in call resynthesis offer promising avenues of research nonetheless, by leveraging existing methods of parametric sound manipulation (Anikin and Herbst 2025) or with generative adversarial neural networks (GAN; Sainburg and Gentner 2021; Beckham et al. 2019). GANs can be especially convenient for this, because unlike parametric methods, they can simply be trained to reconstruct vocalizations without requiring explicit parameterization of the features. GANs are based on a two-part neural architecture: an encoder that compresses the original input (e.g. a spectrogram of a call) into a low-dimensional embedding (e.g. a small vector of values), and a decoder that reconstructs the original input from an embedding. In the case of call resynthesis, the GAN is then trained to compress then reconstruct actual bird calls, as well as generating plausible fake calls from random embeddings. In a study on starlings, a GAN was used to correlate particular features of the original calls with some properties of the embeddings, and resynthesized calls were used in playback experiments to probe how this altered their perception by live starlings (Sainburg and Gentner 2021). Resynthesis thus offers very powerful tools to probe the meaning of particular features in bird calls.

### Focus on segments

Some species are able to produce continuous flows of vocalizations, in a similar manner to how humans can produce long sentences without any pause to take a breath. These continuous sequences often present a significant challenge for researchers attempting to interpret bird vocalizations, as identifying individual units can be more complex. One solution could be to apply analyses similar to how we analyze vocal segments in human vocal production: in all spoken languages, phonemes that are combined into words are organized into two broad classes of sounds segments: (i) the consonants: plosives, transient bursts of energy (e.g.,/p/,/d/,/k/), and (ii) vowels, periodic signals with clear harmonic structure that are typically made with little to no vocal tract obstruction (e.g.,/i/,/u/,/a/) (Hyman 2008).

In their quest to retrace the evolution of human speech, some studies aimed at identifying consonants and vowels in calls of other species (e.g., great apes, Lameira 2018; Grawunder et al. 2022). In birds, Mann and colleagues (2021) were able to efficiently describe budgerigar acoustic productions through the categorization of segments. They designed an algorithm that used identifiable and clear transitions in fundamental frequency, amplitude, and/or spectral dispersion to mark segment boundaries. Like in humans, segments could be clustered into two broad categories akin to vowels and consonants. Moreover, also like humans, budgerigars tended to initiate vocalizations with a plosive segment, while periodic signals were typically found within a syllable (in humans, this is called the ‘CV preference’, Hyman 2008). This change of focus to identify the production units of birds, and in our case of corvids, could very well then lead to new perspectives on where the meaning-bearing units are (Fig. 2). Indeed, budgerigars have syllables that appear to be non-stereotyped and non-repeated (Farabaugh et al. 1992), similarly to how some corvids often lack stereotypy in their calls and songs (Brown 1985; Coombs 1960) and often possess nonlinear phenomena (Fletcher 2000; Anikin et al. 2025). Research on corvid vocalizations may be enhanced by considering these phenomena when identifying segments (see Massenet et al. 2025 for a development on nonlinear phenomena).

### Focus on sequences

When stable units are determined through the presence of calls, acoustic features, or segments, they may however still not be the meaning-bearing units. For example, in human language, meanings are generated through two embedded layers of combinations, also referred to as the duality of patterning (Hockett 1959). Through phonology, meaningless phonemes (e.g.,/h/,/a/,/t/) are combined into morphemes or words (e.g., “hat’). Meaningful words are then combined into hierarchically structured sentences via syntax (Chomsky 1957; Hurford 2007, 2012). The first step is thus to determine if the building blocks of vocal sequences documented in corvids are meaning-bearing units or not, i.e., whether they are functionally analogous to phonemes, or to morphemes. The vocalizations may be meaningless and operate as phonemes whereby only the combination of segments conveys meaning (Fig. 2). For instance, pied babblers combine ‘A’ and ‘B’ calls into AB and BAB sequences (Engesser et al. 2015). Each combination conveys a different meaning, but the B calls are meaningless on their own. The author thus concluded that B calls serve as phonemic contrasts: their presence alters the meaning of the A call. Such phenomena operate constantly in human language, such as when the phoneme/k/is added to the word “at” to create the word ‘cat’ that conveys an entirely different meaning (Engesser et al. 2015). In the case of meaning-bearing units being acoustic features, the same rationale can apply: the presence of one feature alone may not be sufficient to convey a specific meaning, but an aggregation of several features may. For example, in great tits, artificial mobbing calls possessing only one specific acoustic feature (low frequency, noisiness, harmonics, or a large frequency range) were not sufficient to trigger a mobbing response in conspecifics (Salis et al. 2024). However, white noise with a large bandwidth was sufficient to trigger that response (Salis 2022), suggesting that combinations of features may be used to recognize a mobbing call (note that the alternative hypothesis, based on a degree of similarity, still needs to be tested, Salis et al. 2024). Alternatively, if each feature has meaning on its own, then features can be combined to create meaningful aggregations of features; one complex call could be regarded as several layers of features combined to convey a complex meaning (Schlenker et al. 2025). For example, theoretically, an increasing call rate could mean that a predator is approaching, while the frequency range of the call sequence could determine the type of predator (aerial vs ground).

Testing the meaning of combinations can quickly become a daunting task when many single calls are produced, which is often the case in corvids. A large repertoire of calls can lead to a combinatorial explosion when testing combinations. Nevertheless, we encourage future research to expand focus from single calls to vocal combinations since they have the potential to draw analogies to human language principles. Some rudimentary forms of syntactic-like structures have been found in several bird species (e.g., Engesser et al. 2016; Suzuki et al. 2016), including one case in American crows (Richards and Thompson 1978). Given this evidence and the high cognitive capacities of many corvid species it is likely that syntactic-like vocal production is not out of reach for other corvids. Surely, methodological challenges arise when attempting to assess the meanings of call combinations, especially if they are numerous and cannot be singly assessed using classic playback approaches. The development of new analytical tools to assess how the meaning of call combinations is derived from the meaning of the composing units can prove useful here (see section on *call combinations*).

Altogether, these perspectives open new avenues of research for researchers who feel limited by the conventional view that considers calls as meaning-bearing units. This initial step can then be combined with an updated framework on how to correlate vocal units with the specific context of their production. This enhancement of the definition of ‘context’ in animal communication is further elaborated in the section below.

## Improve the classical ethogram-context couple

### Expand and refine what is context

Calls are usually clustered into big categories such as “contact calls”, “food calls”, “alert calls”, describing the broad context in which they are produced and their hypothesized function. One main issue with these classifications is the fact that they often incorporate a large variety of finer contexts that are associated with different communicative needs, resulting in the production of calls that convey different meanings. For example, a call made when discovering food could have a different meaning and function from a call that is made when the flock is already foraging on the food source, but they are usually combined into the large ‘food call’ category. While the call emitted when discovering food may operate to recruit others to the food patch, the calls emitted while feeding together might simply reinforce group cohesion. A second issue is that these broad contextual categories could very well have a meaning that is not directly related to the context itself: a call produced in a ‘food’ context could still have a meaning not directly related to food, such as the production of aggressive calls when competing for food resources.

Two concrete steps can be taken to avoid these broad, uninformative categories. First, there is a strong need to record the study animals in as many different contexts as possible, to ensure that most or all of the vocal repertoire has been sampled. This (time-consuming) effort is crucial to establish which call types, calls with certain acoustic features or combinations of calls, are produced in specific circumstances. Secondly, in order to encompass all possible meanings of utterance, the delineation of the Features of Circumstances (FoC) must be refined (Berthet et al. 2023). External events occurring immediately before, during, or immediately after a given acoustic production should therefore be comprehensively described. These FoC can assist researchers in determining whether an acoustic signal conveys information about a particular *external event* (e.g., the eagle or snake alarm calls described above, see Seyfarth et al. 1980) or about a specific *action intended from the receiver* (e.g., approaching the signaler, Dutour et al. 2019). Moreover, obtaining information about the individual producing the call is essential: call sequences can provide insights into certain *permanent characteristics* of the signaler (e.g., age and sex, Blumstein and Munos 2005) or about its *internal state* (e.g., hunger, high arousal, Leonard and Horn 2001). A large body of work demonstrated how the inner state and in particular the arousal of the caller can influence the type and acoustic structure of the vocalisation produced in a large range of taxa (birds, Osiecka et al. 2023; mammals, Briefer 2012; amphibians, Reichert 2013; and reptiles, Britton 2001) including corvids (Szipl et al. 2017). This comprehensive description of FoC and individual information aligns with the framework proposed by Amphaeris (see Introduction).

To explore these four categories, researchers need to record the most complete set of circumstances surrounding a call. The list of FoC is possibly infinite, but we provide in Table 2a list of categories of FoC with some (non-exhaustive) examples for each category that should provide a thorough exploration of the circumstances surrounding a call. Acquiring all this information simultaneously may be quite challenging, and we recommend first identifying which specific events trigger vocal production in the study species. This will allow obtaining a list of events to link to vocalization that is reasonable (e.g., recent example in chimpanzees of 24 events, including 21 that were documented to elicit vocalizations, Bortolato et al. 2023; Girard-Buttoz et al. 2025). Such a list can be extended on a continuum from the chimpanzee example, possibly less exhaustive but strongly rooted in FoC known to elicit vocalization to attempt to establish a fully exhaustive list (e.g. 336 FoC in a recent bonobo study Berthet et al. 2025). This can however increase the risk of including irrelevant FoC for the study animal and of obtaining a sample size of vocalization insufficient to accurately match the various FoC to the various call combinations. It appears thus crucial to find the right balance between the number of events considered in the ethogram and the expected sample size for a given study.

Concretely, these FoC can be obtained through different sources. First, to identify if a call is triggered by a specific *stimulus*, it is necessary to identify FoC present before the call is produced. The circumstances should include three modalities: what is happening, where and from what. The ‘what is happening’ relates to the perceptual modalities in which the stimulus is produced (movement, sound, odor.). The ‘where’ is related to the position of the stimulus (far or close, on the ground or in a tree, etc.). The ‘from what’ relates to the origin of the stimulus (from a conspecific, a heterospecific, a non-living object…). By precisely listing these circumstances, one can differentiate between alternative hypotheses about the meaning of a given call. For example, for most alarm calls, we do not know whether they signal the presence of a very specific category (e.g., an eagle) or the presence of a non-specific stimulus but at a specific position (e.g., something up) or doing a specific movement (approaching). This can be tested by decoupling the various dimensions of the stimulus, for instance by simulating a novel aerial threat that is not a known predator using a drone to test if animal call production is tied to the presence of the predator itself or to a danger in the sky (Wegdell et al. 2019). Similarly, in the case of food calls, identifying the potentially important FoC to differentiate alternative hypotheses would be useful, such as 1/the presence of different types of food, 2/the quantity of such food, 3/the position of the food, or even 4/the presence/absence of different social members around the caller. In ravens, a basis for such tests already exists : the ‘haa’ calls produced by ravens are produced when food is discovered and vary depending on the social context surrounding the bird (Szipl et al. 2017). The development of new technologies, such as the use of small video cameras with builtin microphones placed directly on the bird (e.g., Thiebault et al. 2016), can tremendously help to match FoC to vocal production, especially for birds that can hardly be followed and observed for several hours.

Secondly, to identify if the call leads to a specific *response from receivers*, we can focus on the set of circumstances that happen just after a vocalization is produced. This includes the precise behavior of conspecifics or heterospecifics and, whenever possible, takes into account the characteristics of these receivers. This approach is commendable and would likely inform on the meaning of a large part of the vocal repertoire of a species but the behavioral response of the caller or the receiver can also operate over long periods of time, and this should not be overlooked (see section on long term effects). In the case of food calls, examining whether these calls are consistently followed by a particular behavioral response from the receivers (e.g., approaching the food source, which, for example, happens after ‘haa’ calls of ravens) could therefore help in determining whether the meaning of the call functions more as a ‘come here’ signal rather than a ‘there is food’ indication. Another approach, inspired by the gestural communication literature (Byrne et al. 2017; Hobaiter and Byrne 2014) could be considered here. In corvid species producing long-flow of vocalizations, observers could note if the same call is repeated several times or if the signaler “elaborate” the signal (e.g., switch to new call types) and precisely determine which behavior in the recipients triggers the end of the vocalization. The underlying assumption here is that a signal (vocal or gestural) aims at modifying the behavior of the receiver and the signal production should stop when the goal has been reached (e.g., when the recipient engages in the desired behavior). Note, however, that this approach is somewhat limited to signals directed at specific individuals in sight of the caller (like gestures) and many vocalizations are broadcasted to the broad (often out of sight) audience and not directed so that every individual capable of hearing the vocalization becomes a potential receiver. In this scenario, monitoring the responses of all receivers, particularly within large groups, may pose a challenge. Research groups possessing birds in aviaries may, therefore, have a comparative advantage in this regard.

Information about the individual calling, although not always clearly observable by an observer in the field, may add crucial information and help posit alternative hypotheses regarding the meaning of an utterance. The *stable properties* of the individual, such as age, sex, size, can be obtained thanks to long-term monitoring projects, with ringed individuals. This is achievable in many wild but also captive corvid populations that have been followed for years and where individuals are identified (e.g. rooks: Martin et al. 2024; ravens: Boeckle et al. 2018; Hawaiian crows: Tanimoto et al. 2017). Secondly, information about the *short-term internal state* of the signaler can sometimes be obtained quite precisely: for example, hunger levels can be modified in captive populations or assessed non-invasively using physiological measurements in their urines (Girard-Buttoz et al. 2011; Palme et al. 2005), emotional states may be inferred from head feather movements in birds (Bertin et al. 2018, 2020), and some information about their health can be derived from microbiome or parasitic analyses from feces or blood samples (Goymann 2005; Townsend et al. 2018a, b; Raidal 2020). Additionally, the short-term internal state of the signaler can be estimated by examining the timing and situational cues associated with the production of a specific call. For example, if a call is produced at a specific time of day, temperature, weather (or any other abiotic factors), we can propose hypotheses about the internal state of the caller in these situations. These hypotheses can, then, be properly tested with experimental procedures. The social environment, taking into account not only the number of conspecifics and heterospecifics in close vicinity, but also their characteristics (age, dominance status, sex, etc.) can also add layers of information. For example, in ravens, the appeasement vocalisations in conflict situations increase if there is the presence of kin, but decrease if by-stander are closely bonded to the aggressor (Szipl et al. 2017). If the same call type, or different call types but with similar acoustic properties, are produced in a range of contexts that appear at first highly dissimilar, it is sometimes important to assess whether there is a common ground across these contexts that could require similar communicative needs (or trigger similar emotions resulting in the production of similar calls). For example, the emotional valence of individuals, and in particular their arousal status, can be similar across contexts e.g., expecting food in a captive setup where individuals are provisioned and receiving aggression are two highly distinct circumstances but generate both emotional valence, which could lead to similar vocal production (e.g., vocalizations containing high proportion of non-linear phenomena, Fournier et al. 2025).

**Table 2:** Categories of features surrounding vocal production that can be taken into account to refine the potential meaning of calls and call combinations

Feature types	Temporality	Category	Examples
**Feature** ** of** ** circumstances** ** (FoC)**	*Immediate* * past*	Social	A conspecific entered the subgroup A conspecific approached
		External stimulus	Food, Predator
	*Present*	Caller activity	Feed, Rest, Travel
		Social	Give/receive aggression Is approach/being approached Allopreening
	*Immediate future*	Activity	Caller/conspecific changes activity
		Social	Caller/conspecific leaves Caller/conspecific approaches Conspecifics stop fighting
	*Mid-term future* * (several minutes)*	Social	A conspecific enters the subgroup/the caller joins a new subgroup, after several minutes
**Caller** ** attributes**	*Permanent*	Physiological	Sex
	*Current*	Physiological	Age, Size
		Social	Dominance rank, Position in the social network
**Caller** ** internal** ** state**	*Current*	Valence	Positive valence (e.g., affiliation, expecting food) Negative valence (e.g., aggression)
		Arousal	High arousal (e.g., aggression, food) Low arousal (e.g., rest)
		Physiological status	Hunger, Stress

### Include multimodality

When attempting to comprehend the meaning of a vocal signal, it is crucial to consider that vocalizations are often produced concurrently with signals from other modalities, such as facial expressions and gestures. The simultaneous or sequential integration of signals from several modalities is referred to as multimodal signaling (Waller et al. 2013). It is now widely acknowledged across a broad range of taxa (primates: Slocombe et al. 2011; Fröhlich et al. 2019; arthropods: Partan and Marler 1999; songbirds: Cooney et al. 2018) that studying each signaling modality in isolation limits the ability to fully describe an animal communication system and may result in incorrect or unclear meaning attribution of specific signals. Combined signals from different modalities can be redundant, serving the same function, and simply enhancing the receiver’s response when produced together (Smith and Evans 2013). In this case, disregarding signals from other modalities when assessing the meaning of vocal signals may not impair the results. In contrast, when signals from multiple modalities are not redundant (Smith and Evans 2013) so that one modifies the meaning of the other signal or only the combination of both signals convey meaningful information (Partan and Marler 1999), then the risk of attributing the wrong meaning to a vocal signal or of not being able to attribute any meaning at all can be significant. A classical approach here is to test or map the meaning of each single signal separately and then assess the meaning of the combination (Smith and Evans 2013). The way the meaning of the combination is derived from the meaning of each single signal can then be assessed using newly developed methods to calculate distance in the semantic space of each signal and their combination or go-no go experiments (Elie et al. 2025) (detailed in the following sections). Pertaining to corvid communication, future research embracing a multimodal approach, as is done in communication studies in other taxa (e.g., in primates: Fröhlich et al. 2019; Slocombe et al. 2011), may be of interest, as potentially - but not conclusively - suggested by a few studies in ravens (Pika and Bugnyar 2011; Van Rooijen 2015; Pika 2016) and some observations in other species (e.g. rooks Coombs 1960; jackdaws: Katzir 1983; magpies: Baeyens 1979).

### Consider long-term effects of vocalizations and socially dependent meanings

Several other factors can complicate the task of attributing meanings to vocalizations, often requiring further assessments beyond considering just the immediate features and circumstances surrounding signal production in observational studies or the immediate reaction of the target in playback experiments. Vocalizations can elicit behaviors in recipients that become evident only after extended periods of time. For instance, in primates, bonobos produce high-hoot vocalizations that are audible at least 700 m away and are frequently used to maintain auditory contact and coordinate movements with other subgroups. Bonobos can add a whistle to the high hoot to form a whistle high-hoot combination that indicates their motivation to join another subgroup (Schamberg et al. 2016). However, this behavioral response can occur up to 15 min after the call production, necessitating “long-term” monitoring of the caller’s behavior to infer the function of the call.

In addition, in some cases, the immediate behavioral reaction or the set of features surrounding vocal production are not enough to determine the meaning of a call not because its effect is spaced in time but because the call functions to prolong ongoing activity. Chimpanzees for instance produce a hoo vocalization that prolongs the time individuals rest cohesively in the same place, so that this vocalization has been interpreted as meaning “stay put” (Bouchard and Zuberbühler 2022). Here, it is the absence of behavioral changes that indicates the meaning of the call.

The recipient’s behavioral response to vocalizations can also be influenced by past events and the caller’s identity. Chimpanzees live in large multi-male multi-female groups and form long-lasting equitable social bonds with specific individuals with whom they frequently form coalitions to outcompete conspecifics (Mitani 2009). A playback experiment tested the ability of chimpanzees to monitor third-party relationships. A focal chimpanzee was observed for hours until it engaged in aggression with a conspecific, and, 90 min later, an aggressive bark was played back either from an individual closely bonded to the previous opponent or from a non-bonded individual (Wittig et al. 2014). Chimpanzees largely ignored the bark from non-bonded individuals to the previous opponent but paid great attention to the bark of individuals strongly bonded to the previous opponents, often moving away from the speaker. This example highlights the importance of considering long-term social contexts and interaction history when interpreting behavioral reactions. In crested macaques, playback experiments of alarm calls given to pythons showed that individuals were more likely to attend to the calls of closely bonded individuals (Micheletta et al. 2012), illustrating further the importance of considering the relationship between the call provider and the recipient when inferring vocalization meanings. For corvid species living in large flocks with high degrees of fission-fusion dynamics (Clayton and Emery 2007) and differentiated social relationships, complex audience effects have indeed been observed (e.g., in ravens, Szipl et al. 2017). Apart from its semantic implications, comparative research could facilitate the identification of similar cognitive processes involved in communication across clades as distantly related as birds and mammals. When possible, we recommend investigating the meaning of vocal signals in habituated populations with long-term behavioral monitoring to assess social relationship strength. Following specific target birds for as long as feasible to record vocal production in temporal, social, and environmental contexts, can allow the assessment of long-term triggers and effects of vocalizations.

### Consider sequential vocal exchange rather than isolated vocalizations

While many vocalizations can trigger short—or long-term behavioral reactions on their own, some only operate effectively when part of a vocal exchange between individuals or as part of a multi-individual sequential vocal production. In the bonobo whistle-high hoot example, the caller travelled towards another subgroup almost exclusively if they heard a vocal response from the target subgroup (Schamberg et al. 2016). Likewise, in the chimpanzee hoo example, individuals who answered to the first hoo by another hoo rested for longer (Bouchard and Zuberbühler 2022). Not considering vocalization as part of an interaction in those studies would likely have prevented the authors from assessing the function of the vocalizations. As in bonobos, coordinating group movement is a challenge faced by most group-living animals. In several species, from diverse taxa (meerkats: Bousquet et al. 2011; wild dogs: Walker et al. 2017; and gorillas: Nellissen et al. 2024), recently including corvids (Dibnah et al. 2022), this challenge is resolved using the sequential production of vocal signals from several individuals in what has been labelled a ‘quorum response’. Here, an individual signals its intention to travel in a certain direction, but the group movement is initiated only if a certain fraction of the group members reply to this vocalization by producing their own vocalization through consensus-building dynamics. Such group effects are important to consider when establishing the meaning of a vocalization since the absence of behavioral response might not mean that the vocalization is meaningless or does not function to modify the receivers’ behavior but rather that its function is solely expressed when part of a multi-individual sequence.

## Determine meaning

### Playback experiments

In the study of animal communication, playback experiments are seen as the gold standard to establish the meaning of animal calls. This approach allowed to identify various call meanings in corvids such as predator related calls [alarm (Bílá et al. 2017; Davídková et al. 2020), recruitment to mobbing (Griesser 2008, 2009) and sentinel calls (Bednekoff et al. 2008)], food related calls (Bugnyar et al. 2001), contact calls (McCaig et al. 2015; Munteanu et al. 2009), copulation calls (Gill et al. 2020; Hooper et al. 2024), and calls that facilitate group movement coordination (Dibnah et al. 2022).

This two-step process begins with naturalistic observations allowing to map calls to specific events by documenting the context in which vocalizations are emitted or the response they elicit. In the second step, playback experiments test whether receivers respond as predicted in the absence of the original context and confirm the hypothesised call meaning. These playback tests can show that the behavioral response is triggered simply by the vocalization and is stimulus independent, indicating that specific information can be decoded from the vocalization by the receiver (Dezecache and Berthet 2018; Evans 1997; Macedonia and Evans 1993). Such a method is also useful to explore promising concepts such as the notion of compositionality (i.e. ‘*a process by which meaning is determined by the meanings of the constituent parts and the rule that combines them*’, Zuberbühler 2020), a key component of human communication, possibly found in distant clades such as birds (Suzuki et al. 2016, 2017). In corvid, most playbacks studies focused on predator related calls (reviewed in Wascher and Reynolds 2025). While many confirmed the call type meaning suggested by observations, some fail to validate all tested call types. For instance, in Torresian crows, playbacks confirmed the meaning of contact, flee alarm and begging calls but not the mobbing function of one call type, as receivers did not approach the speaker as expected (McCaig et al. 2015).

Playbacks have their own limitations. They often rely on a narrow range of measurable behavioural responses, mostly limited to approaching/avoiding the speaker, gaze frequency and duration and vocal reply. This limitation is nicely illustrated by a foundational study in 1971 on common crows. This study tested the meaning of five call types using playbacks but predicted the same behavioural response (moving towards the speaker) for four of them, making it impossible to distinguish between potential meanings (Chamberlain and Cornwell 1971). These constraints become even more apparent when studying call combinations (see below).

### Matching circumstances to utterance types to provide insight into the meaning of call combination

While the vast majority of animal communication research has traditionally focused on analyzing the meaning and function of single calls and songs, the past 15 years have seen a significant shift, particularly in primate studies, towards examining the meaning of call combinations. These studies have provided valuable insight into the evolutionary origins of human language, which is inherently combinatorial (Engesser and Townsend 2019; Suzuki et al. 2020; Townsend et al. 2018a, b; Zuberbühler 2019). They also were able to evaluate how animals use combinations to expand the limited range of meanings conveyed by single calls only (e.g. Girard-Buttoz et al. 2025).

Playback experiments have revealed various scenarios via which the meaning of single calls is altered when combined with other calls into a call combination. In compositional combination, the meaning of the combination is derived from the composing calls, either by combining the meaning of both calls (e.g., alarm + recruit call combinations in pied babblers and Japanese tits, Engesser et al. 2016; Suzuki et al. 2016) or through affixation where one call modifies the meaning of the other (e.g., adding a “oo” to the alarm calls “krack” and “hock” diminishes the urgency of the message in putty-nosed monkeys, Ouattara et al. 2009). Call combinations can also generate new meanings that are not related to the meaning of the composing calls (e.g., alarm call 1 + alarm call 2 = travel call in putty-nosed monkeys, Arnold and Zuberbühler 2006; rest call + affiliative call = nest call in chimpanzees, Girard-Buttoz et al. 2025). Although for most species at most one or two short two-call combinations (bigram) have been reported, often limited to alarm context, recent studies in great apes and marmosets highlight the potential of certain species to utter a large diversity of call combinations across a large range of daily life events (Berthet et al. 2025; Bortolato et al. 2023; Bosshard et al. 2024; Girard-Buttoz et al. 2022).

**Fig. 4:**
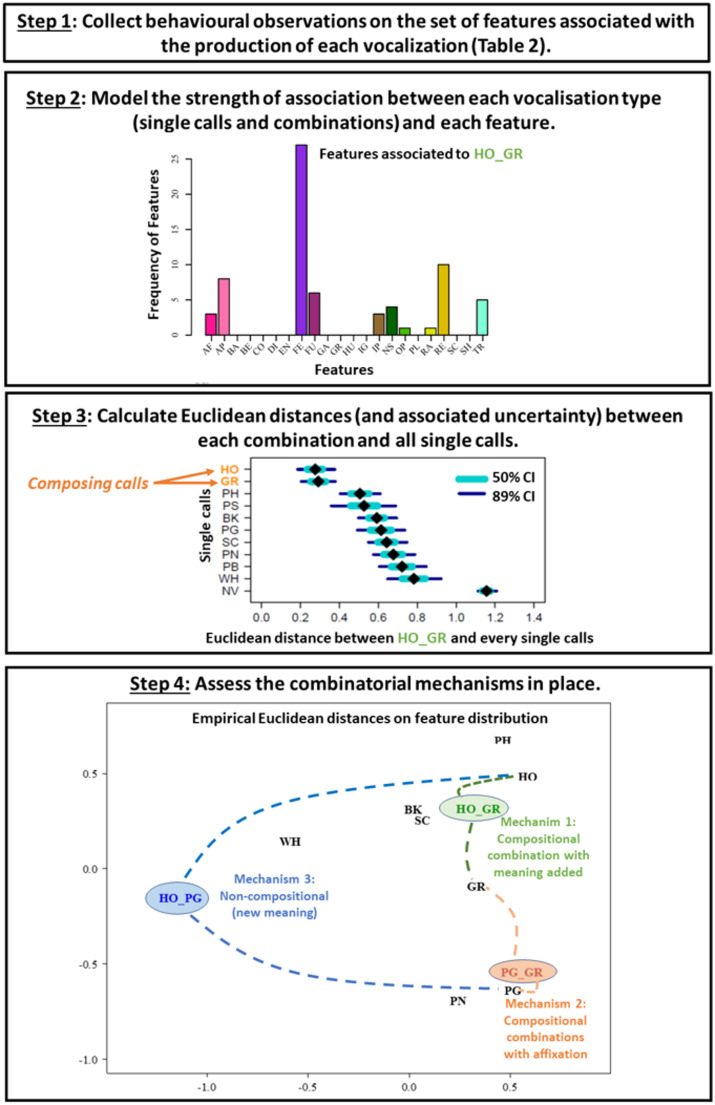
Steps to assess the meaning of call combinations following the feature-to-vocalization matching approach. Graphical examples are derived from empirical data on wild chimpanzees (refer to Girard-Buttoz et al. 2025 for details). Step 1 consists of recording a fine-tuned, clearly defined set of features surrounding each vocalization (see details in section on FoC and Table 2). Step 2 consists of quantifying and modelling the frequency of association between each feature and each vocalization type. The example here shows the frequency distribution of the features associated with the chimpanzee call combination HO_GR, composed of a ho (HO) and a grunt (GR). Step 3 consists of using the posterior distribution from a Bayesian model based on the behavioral observation to calculate Euclidean distances between each call combination and each single call. The Figure here represents the Euclidean distance (and associated CI) between the combination HO_GR and every one of the single calls in the chimpanzee repertoire. The composing calls (HO and GR) are indicated in orange. In this example, HO_GR is closer in terms of Euclidean distance to HO and GR than to all the other calls. Step 4: Establish which combinatorial mechanism operates for each combination. Examples are provided of three mechanisms operating in chimpanzee bigrams. Scenario 1: compositional combination with meaning added since HO_GR is closest to the composing calls (HO and GR) and equidistant to them (see also the Figure in step 3). Scenario 2: compositional combination with affixation, since this PG_GR is much closer to one of the composing calls (PG) than the other. Scenario 3: Non-compositional combination (new meaning) since HO_PN is far from all single calls

These *full repertoire approaches* rely on documenting the full vocal sequence repertoire of species across all daily life events, allowing them to evaluate all the combinatorial mechanisms (mechanisms via which meaning is altered when calls are strung into combinations) used by a species. These methods can be adapted to both captive and free-ranging animals, even if individual identity cannot be obtained. For instance, these studies have revealed that chimpanzees and bonobos use all combinatorial mechanisms described above across a diversity of call combinations and situations (Schamberg et al. 2016; Berthet et al. 2025; Girard-Buttoz et al. 2025). Future studies on corvid vocal communication should embrace a full repertoire approach to uncover the vocal capacity of each species. Assessing the meaning of each call combination using traditional playback approaches can, however, prove an impossible task, especially for species that use many combinations, or with highly graded vocalizations, like corvids. Recent methodological developments allowing for mapping the feature of circumstances (FoC) surrounding vocal production to each single call and combination have proven useful (Berthet et al. 2025; Girard-Buttoz et al. 2025). This approach requires detailed documentation of the features surrounding each vocal production (see section on FoC above and Table 2). Bayesian multinomial models can then be applied to these datasets (see e.g., Girard-Buttoz et al. 2025) with the utterance type (single call or combination) as the predictor (as a random factor) and the features of circumstances (FoC, see Berthet et al. 2023, 2025) the response. Here, the FoC are entered as a matrix with 0 s and 1 s, and a given utterance can have more than one FoC associated with it (e.g., greeting an individual in a food patch after a fusion between subgroups). Such a model allows for controlling for repeated sampling of the same individuals. Posterior distribution of the proportion at which each feature is associated to each utterance type can then be extracted and used to calculate Euclidean distances between each utterance type in terms of FoC distribution and their associated uncertainty. This approach allows for quantifying statistical support for different combinatorial mechanism scenarios (Fig. 4): Scenario *1*: Compositional combination with meanings added (AB = meaning of A + meaning of B) is characterized by a strong posterior support for the Euclidean distance between AB and the composing calls A and B being shorter than between AB and all other single call types. *Scenario 2*: Compositional with affixation (AB = Aa) is characterized by AB being closer to A than to all other single calls (but still not identical). *Scenario 3*: Non-compositional combinations where the combination AB generates an entirely new meaning (AB = C) unrelated to the meaning of the composing calls (Fig. 4). Here, the distance between AB and all single calls (including the composing call) is expected to be large, and no particular call should have a statistically meaningful shorter distance to AB. This approach also allows testing ordering effects on meaning by comparing the meaning of AB with that of BA, a crucial step in determining if animal call combinations are syntactic (Suzuki et al. 2016). To our knowledge, the ability to attribute different meanings to combinations of the same calls in different order have not been demonstrated in corvid. This might however not be out of reach for them since they react differently to a sequence of two calls uttered by two different individuals and its reverse during hierarchical vocal interactions (Massen et al. 2014).

### Determine meaning using a go-no go experimental paradigm

The approaches described above offer valuable insights into the potential meaning of each utterance type from the signal production side of the multifaceted concept of meaning (see introduction and Amphaeris et al. 2023). However, these approaches do not inform us about how different utterances are understood by receivers. An important next step thus is to determine whether the degree of differentiation between the meanings of various utterance types, as derived from behavioral observations, aligns with the mental categorization by receivers.

As previously mentioned, classic playback experiments have been used to assess the meaning of a fraction of the vocal repertoire of many species, primarily alarm-related calls, leaving much uncertainty about most of the calls produced. For example, despite 65 years of study, the meaning of less than half of the call types in the chimpanzee vocal repertoire has been assessed using playbacks (Crockford 2019), and only one call combination has been tested (Leroux et al. 2023).

Recently, a novel and ingenious methodological approach was proposed (Elie et al. 2025) to alleviate the practical limitation of testing the meaning for the receiver of each call type using a separate playback design each time. This method is based on a “Go/No-go” experimental design, allowing for the testing of the categorization of call types and their similarity in meanings for the receiver. This approach enables the testing of an entire species’ vocal repertoire within a single experimental design, including call types such as resting calls or contact calls, for which the suspected meanings make it difficult to predict specific behavioral responses using classic playback experiments. In fact, if a rest call is played back to an individual, the recipient is not expected to exhibit any behavioral reaction, even if the meaning of the call is processed as expected.

This “Go/No-go” approach is based on a captive experimental design, where a sequence of a specific call type is played back to the recipient. At the end of the sequence, the animal receives a reward if the call type played back corresponds to the category of call types being tested. The animal can interrupt the playback before it ends to trigger a new playback if they perceive the call type as belonging to the wrong category. This setup allows for the assessment of error rates based on correct and incorrect classification of different vocalizations as belonging, from a human perspective, to the same or different call types. The error rate indicates which call types are processed by the animal as having similar meanings and the degree of difference in meaning perceived by the animals for the various call types.

Such an approach was used in zebra finches, utilizing 6-second stimuli, which demonstrated that they classified their 11 call types in their repertoire, as established by humans through acoustic analyses, with low error rates. However, a fine-tuned analysis of error rate revealed that the birds were more likely to confuse two different call types classified based on behavioral observations as having similar meanings and categorized in the same semantic hyper-category (e.g., contact calls, agonistic calls) than where the meanings differed greatly (Elie et al. 2025). This confirmed that the birds processed the semantic content of different call types as categorized based on naturalistic observations. Interestingly, in this study, semantic content similarity explained the error pattern in the Go/No-go experiment better than the acoustic distance between call types.

Although this experiment focused solely on single calls, it opens promising avenues for addressing questions related to the meanings of call combinations for receivers, and how they are derived (or not) from the meanings of the component calls. Since previous cognitive tests on corvids have demonstrated their proficiency in Go/No-go experimental design (e.g. Brecht et al. 2019; Liao et al. 2024), it is highly likely that such experimental procedure would be a practical solution for testing the meaning for the receiver of corvid vocalizations. This is particularly relevant for corvids, where many captive or semi-free-ranging populations already exist from pre-existing cognition research, and experimental designs can be implemented in test chambers.

### Use cognitive tests to understand communication processes

Assigning meaning to a specific unit is an internal process. As a consequence, one way to approach meaning is to understand the hidden cognitive mechanisms implicated in communication, and more generally, in how animals comprehend their surroundings. Cognitive tasks linked to complex communicative systems are numerous, and have been explored in several species, including primates, birds, and insects. For example, researchers have demonstrated that disjunctive syllogism (i.e., the ability to process the logic: A or B; not A, therefore B) is present in apes and parrots (Pepperberg et al. 2019; Ferrigno et al. 2021). Similarly, bees are able to cardinally order natural numbers, including zero (Howard et al. 2018), and a study has shown that two species of birds (budgerigars and zebra finches) do not perform in the same way when facing the same artificial grammar experiments (Spierings and ten Cate 2016).

With a similar line of thought, cognition and language properties can be linked through the Language of Thought hypothesis. The Language of Thought theory posits that mental representations are structured similarly to sentences; they are composed using symbols akin to those found in formal languages (Fodor 1975, 2008). This organization allows us to construct complex mental structures from a limited set of primitive operations. This Language of Thought hypothesis may assist in uncovering the mechanisms underlying meaning in animals, as it allows researchers to explore how animals can concatenate elements to form novel, larger constructs. Showing that some species have the capacity to compositionality cluster elements, even outside acoustic communication systems, can provide important information about their capacity to do so in an acoustic system. Such projects are currently explored mainly in nonhuman primate species. For example, baboons were able to understand negative representations such as ‘not blue’, showing that non-human primates have the ability to combine abstract concepts like humans do (Dautriche et al. 2022). Developing such protocols across a broader range of species could prove essential in establishing an integrated theory of communication, and research on corvid cognition represents one of the most promising avenues in avian studies. In fact, numerous cognitive tests and trained bird subjects are already available. For instance, corvids have been included in experiments suggesting their potential capacity for recursion, a mechanism traditionally considered to be uniquely human (Liao et al. 2022; although see Rey and Fagot 2023 for critics). Concentrating on the LoT theory may expand the current understanding of the evolution of language in animals, and corvid species are particularly suitable candidates for investigating these primitive, foundational operations necessary for complex communication systems.

### Develop formal models

Formal linguistics employs formal methods (e.g., from logic and formal language theory) in the analysis of natural languages. The goal is to have fully explicit and hence testable theories. When proposing a semantic formal model, linguists derive a set of logical rules and principles to formally describe the meaning of a unit. Applied to animal call sequences, this method allows for the formulation and test of explicit novel hypotheses, which is important when trying to understand the core meaning of utterances. Importantly, the goal of such a method is not to directly compare non-human systems to human communication, but to use an efficient methodology to analyze ecological findings and propose useful alternative hypotheses (Schlenker et al. 2022). Initial examples of formal models of animal calls have been documented in primates (Schlenker et al. 2016) and birds (Schlenker et al. 2023, 2024). For example, in Japanese tits, formal analyses have demonstrated that two alternative hypotheses can be employed to interpret the meaning of the ABC-D combinations (Schlenker et al. 2023). These models enable us to determine the expected outcomes if non-trivial compositionality is present, as opposed to trivial compositionality: in the case of non-trivial compositionality, the meanings of the two parts (ABC = ‘something that licenses alert’, and D = ‘something that licenses recruitment’) are combined so that the overall ABCD call signifies that the *same object* licenses both recruitment and alert behaviors, which consequently facilitates anti-predator mobbing responses. In formally describing this theory, the authors were thus able to derive a series of predictions that now warrant empirical testing in the field. This kind of exchange can lead to follow up experiments, such as the one providing a stronger argument towards non-trivial compositionality in this species (Suzuki and Matsumoto 2022). This new experiment demonstrated that tits did not respond if the ABC and D calls were played in sequence but from two different locations, reinforcing the conclusion that the combination is non-trivial. Although such exchanges are currently limited, balanced discussions between animal behavior researchers and linguists hold significant potential to provide new insights into corvid communication.

### Focus on large evolutionary scale

Finally, a valuable approach to changing perspective on the meaning of a given unit is to broaden our perspective by conducting large-scale inter-species comparisons (Schlenker et al. 2022). The presence of large audio recording databases and efficient exchanges between international teams now enable researchers to conduct comparative research with greater effectiveness than ever before. Comparative research on the meaning of calls is however currently largely underexplored. First projects have unraveled the impressive age of specific calls (boom calls of primates, Schlenker et al. 2016) or even ordered-constrained combinations (F-D call in all Paridae, Salis et al. 2025). These reconstructions can help the development of novel hypotheses about their emergence and the evolution of their meaning. This large-scale comparative approach could be used with a specific focus on meaning. For example, the relative categorization of alarm calls (producing only one broad call, or several specific calls) could be traced back and discussed with an evolutionary perspective (Thouzeau et al. 2025; in press).

Another approach to investigate meaning on a larger evolutionary scale may be interspecific communication, i.e. testing what is conserved in the equivalent calls of different species (or populations). For instance, corvids have been shown to react to alarm calls from other corvid species but not non-corvids (Davidková et al. 2020). This approach must be carefully considered due to the open-ended vocal learning abilities of corvids, including heterospecific mimicry: comparing sympatric species may not be conclusive, as individuals may well learn to mimic some calls across the species barrier. Instead, testing allopatric species, or at least allopatric populations of partially-sympatric species that cannot be in vocal contact, may offer insight into what acoustic structures and meanings are conserved across the evolution process.

Overall, in corvids, such comparative studies largely remain to be done, in no small part due to many species lacking established repertoire to compare in the first place. Efforts to cross-examine already-recorded data and coordinate future research have started, as evidenced by this very issue, and could yield promising results in the coming years.

## Conclusion

In conclusion, corvids are celebrated for their exceptional cognitive abilities and, in many cases, extensively complex social lives. According to the social complexity hypothesis for communicative complexity (SCHCC), they should also possess complex communication systems to navigate their social lives. The meaning of corvid calls hasn’t been extensively explored yet because of some practical limitations, in part due to the complex acoustic properties of corvid vocalizations. Nevertheless, modern methods of computational bioacoustics and linguistics have been developed and applied to various other species with some of the same challenges as corvids. One remaining limitation is the lack of recorded data, and especially the lack of integration between what already exists, which hinders comparative research (for instance, many research teams record data from only one species, rarely more, often with their own annotation scheme or software that can easily be incompatible with that of another team). In this review, we describe three aspects that may guide future research in corvid communication: changing the way we perceive meaning-bearing units, defining better the context surrounding a call, and adapting promising methods tested on non-corvid species. We hope this will foster new comparative research initiatives and promote productive collaboration between teams and disciplines that may have, traditionally, developed independently.

## Data Availability

No datasets were generated or analysed during the current study.
